# A fine structure genetic analysis evaluating ecoregional adaptability of a *Bos taurus* breed (Hereford)

**DOI:** 10.1371/journal.pone.0176474

**Published:** 2017-05-01

**Authors:** H. D. Blackburn, B. Krehbiel, S. A. Ericsson, C. Wilson, A. R. Caetano, S. R. Paiva

**Affiliations:** 1 National Animal Germplasm Program, Agricultural Research Service, USDA Fort Collins, CO, United States of America; 2 Department of Animal Science Colorado State University, Fort Collins, CO, United States of America; 3 Sul Ross University, Alpine, TX, United States of America; 4 EMBRAPA—–Recursos Geneticos e Biotechnologia Brasilia, BR; 5 CNPq Fellow, EMBRAPA LABEX, Fort Collins, CO, United States of America; University of Illinois, UNITED STATES

## Abstract

Ecoregional differences contribute to genetic environmental interactions and impact animal performance. These differences may become more important under climate change scenarios. Utilizing genetic diversity within a species to address such problems has not been fully explored. In this study Hereford cattle were genotyped with 50K Bead Chip or 770K Bovine Bead Chip to test the existence of genetic structure in five U.S. ecoregions characterized by precipitation, temperature and humidity and designated: cool arid (CA), cool humid (CH), transition zone (TZ), warm arid (WA), and warm humid (WH). SNP data were analyzed in three sequential analyses. Broad genetic structure was evaluated with STRUCTURE, and ADMIXTURE software using 14,312 SNPs after passing quality control variables. The second analysis was performed using principal coordinate analysis with 66 Tag SNPs associated in the literature with various aspects of environmental stressors (e.g., heat tolerance) or production (e.g., milk production). In the third analysis TreeSelect was used with the 66 SNPs to evaluate if ecoregional allelic frequencies deviated from a central frequency and by so doing are indicative of directional selection. The three analyses suggested subpopulation structures associated with ecoregions from where animals were derived. ADMIXTURE and PCA results illustrated the importance of temperature and humidity and confirm subpopulation assignments. Comparisons of allele frequencies with TreeSelect showed ecoregion differences, in particular the divergence between arid and humid regions. Patterns of genetic variability obtained by medium and high density SNP chips can be used to acclimatize a temperately derived breed to various ecoregions. As climate change becomes an important factor in cattle production, this study should be used as a proof of concept to review future breeding and conservation schemes aimed at adaptation to climatic events.

## Introduction

Climate change (CC) will have broad and comprehensive effects upon agriculture and food security as documented by the Intergovernmental Panel on Climate Change [1] and will require adjustments in livestock management and breeding strategies to maintain and perhaps increase food security. Increased frequencies of extreme heat stress, such as the 2003 heat wave in France which was three standard deviations above typical temperatures [2], suggests work must be initiated to adapt livestock to CC. Specifically to livestock, the impact of heat stress on production (e.g., growth rates, reproduction, milk production) has long been of interest to the livestock sector due to its economic ramifications [3, 4, 5, 6].

Some authors have suggested a substantial realignment of species raised by livestock producers as a response to forage, disease, and ambient temperature stresses caused by CC [7]. However, such a shift would likely result in significant capital losses to livestock producers. Similar arguments suggest shifting from *Bos taurus* breeds to *Bos indicus* breeds, and particularly breeds from developing countries that are believed to already be better adapted to environmental stresses [8] like heat and associated diseases, might be a solution. While it is recognized that *Bos indicus* breeds exhibit higher levels of tolerance to heat stress when compared to *Bos taurus* breeds a number of disadvantages in productivity and quality traits, such as delayed puberty, lower vigor of newborn calves, lower growth rates and carcass quality [9, 10, 11] have been reported.

The Bovine HapMap Consortium [12] reported a general contraction of genetic diversity among cattle breeds in the last century. Such contractions might potentially impact breeders’ abilities to select within breeds for traits that may adapt cattle to CC variables. O’Neill et al. [13] provided examples of how *Bos taurus* breeds have been adapted to subtropical and tropical environments under natural and artificial selection, thereby improving their resilience to heat stress. N’Dama (Africa), Senepol (Virgin Islands) and more recently the Adaptaur (Australia) [14] are examples. Additional comparisons among *Bos taurus* beef breeds (Angus, Hereford, Senepol, and Romosinuano) have been performed [15] and observed differences illustrated genetic variability is present and available for use. Substantial work exploring within breed variability of Holstein dairy cattle has been performed [16, 17] and results suggests available genetic variability could be used to achieve genetic gains in heat tolerance in the breed. For Herefords in a preliminary phenotypic evaluation of weaning and yearling weight differences among ecoregions were found (Stacey Sanders personal communication). To further explore this finding, this study was performed using a wide sampling of Hereford cattle as the breed is widely distributed across US ecoregions and it is found in 59 developed and developing countries [18]. The objective of this study was to evaluate the genetic diversity of Hereford (*Bos taurus*) among the ecoregions where the cattle were produced, with specific focus on SNPs associated with an animal’s ability to cope with environmental stressors that may confer an ability to adapt to climate change variables. Having such an assessment can be a useful proof of concept in determining this breed’s potential ability to respond to CC, and therefore provide insights on how breeders may wish to utilize currently available genetic variation to achieve particular breeding objectives.

## Materials and methods

### IACUC review

No samples were collected for this study; rather they were collected as part of other studies or program activities not associated with this study. To provide an overview of the samples we detail the following. Cattle blood and semen samples were collected by individual livestock owners and submitted to various research entities for germplasm collection or genomic analysis. These private businesses have no obligation to comply with IACUC protocols as the cattle were their property. The majority of the samples were submitted to the American Hereford Association for genomic analysis. The Association worked with the ARS in performing the genomic analysis. The remaining samples were acquired from cattle breeding companies by the ARS National Animal Germplasm Program as part of their efforts to conserve genetic resources.

### Classification of ecoregions

**Fig 1:**
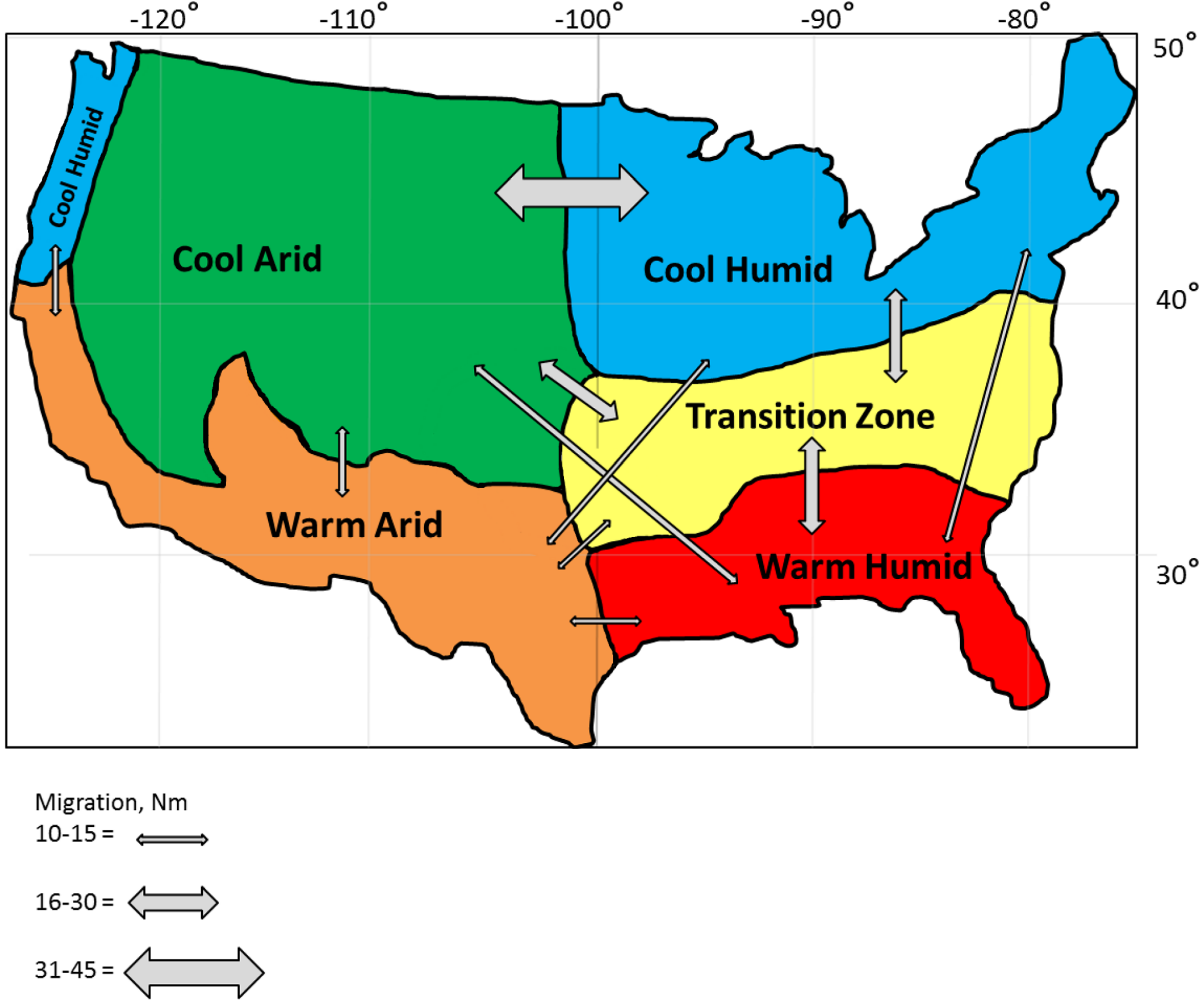
Ecoregions for the United States and the relative magnitude of gene flow (number of animals migrating between ecoregions per generation) among the ecoregions as indicated by the thickness of the arrows.

Classifying climatic zones where livestock are raised better enables our understanding of how environmental conditions can impact productivity [19, 20]. A widely used system for globally classifying climates was proposed by Koppen-Geiger and has been revised [21]. Taking such approaches into account we partitioned the US into five areas: cool arid (CA), cool humid (CH), transition zone (TZ), warm arid (WA), and warm humid (WH: Fig 1). The delimited areas were based upon the climatic features: annual precipitation, summer temperatures, winter temperatures and summer humidity (Table 1).

**Table 1:** Climatic variables for five ecoregions within the United States.

Ecoregion (abbreviation)	Average annual precipitation, mm	Average summer temperature, °C (range)	Average winter temperature, °C (range)	Average summer humidity, %
Cool Arid (CA)	373.6	21.0 (18.7–22.6)	-2.1 (-2.84–-0.63)	32.9
Cool Humid (CH)	999.0	21.2 (19.7–22.4)	-3.0 (-4.5–-2.2)	53.9
Transition Zone (TZ)	913.4	24.9 (23.6–25.8)	2.9 (1.8–3.8)	53.4
Warm Arid (WA)	297.7	28.9 (27.7–29.6)	11.2 (10.4–12.7)	34.7
Warm Humid (WH)	1305.6	27.3 (26.5–27.9)	10.1 (9.0–11.0)	57.2

Climate data for the delimited ecoregions was obtained from the National Oceanic and Atmospheric Administration [22, 23, 24] and were used to estimate thermal stress. Calculating the temperature humidity index (THI) [23, 24], monthly measurements of afternoon average relative humidity (RH) from 1938–2014 and normal daily maximum temperatures (1981–2010) were acquired for every state within the five ecoregions. Because highest temperatures typically occur in the afternoon, afternoon RH was utilized to calculate a peak measurement of heat stress. Climate variables were obtained from the nearest weather station to the Hereford breeder whose animal(s) had been genotyped. Climate variables from states that did not contain Hereford breeders were obtained from the most central weather station to represent that state’s weather. Within each region, daily temperature and humidity values were used to formulate an average monthly THI. The temperature-humidity Index (THI) has been formulated to represent the combined effects of thermal stress and was calculated [25]:

THI=(1.8T+32)−((0.55−0.0055RH)(1.8T−26))

where: T = temperature in Celsius; and RH = relative humidity.

**Fig 2:**
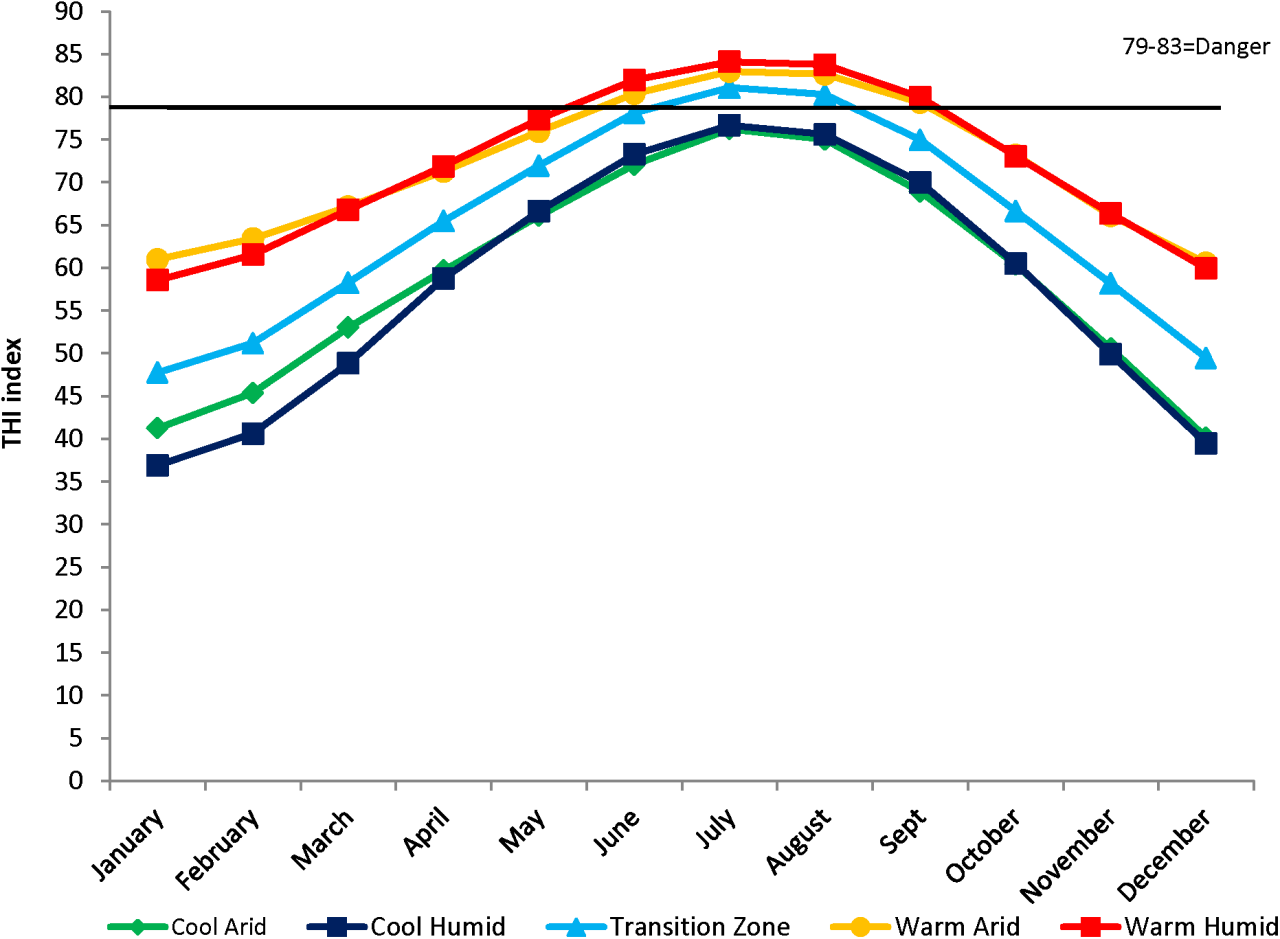
Average monthly thermal heat index by ecoregion, where the horizontal line denotes the threshold for the danger category.

The average monthly THI was then used as an indicator of the level of heat stress an animal might encounter during any month using the classification of: normal: <75; alert: 75–78; danger: 79–83; and emergency: >84 [26]. The period between May and September was the focus of the analysis as these are the hottest months of the year and when the average monthly THI was at or above the “danger” category (Fig 2).

### Animal genotyping and evaluation

There has been previous research on Hereford documenting the extent of genotype-environmental interactions in cool arid and warm humid environments for a number of characteristics and their ability to adjust to new environmental conditions [27, 28]. More recently inbreeding levels of 10 to 18% and an effective population size of 85 for the U.S. Hereford population were reported [29].

Data from 577 Herefords genotyped with either 50K or 777K Bovine SNP chips (Illumina Inc. CA, US) were acquired and initially evaluated. A total of 491 samples were part of the “2,000 Bull Project” [30] where each respective breed association was responsible for choosing bulls believed to be representative of the breed. A subpopulation of animals from that study included 180 bulls from Line 1, an inbred population of Herefords [31]. High-density (777K) data from 86 animals, broadly sampled from multiple geographic locations and part of the national gene bank were also part of this study. Data from Nelore (n = 47) cattle (*Bos indicus*), genotyped with the 777K high density SNP chip, were acquired from US gene bank or Brazil [32] were used for comparative analysis with Hereford samples from WA and WH ecoregions.

The preliminary working data set was formed by using all Hereford samples mentioned. Data were pruned to 45,066 SNP markers common to all samples and converted to A/B format following the recommendations from Illumina Inc. (http://www.illumina.com/documents/products/technotes/technote_topbot.pdf) to guarantee uniformity in the reporting allele frequency calls. Additional quality controls were carried out to exclude samples with call rates (CR) < 0.85, Minor Allele Frequencies (MAF) < 0.05, Hardy Weinberg Equilibrium (HWE) < 0.001; and linkage disequilibrium (LD) r^2^ > 0.5 in 50 marker windows. The final data set for genetic structure study was carried out with 14,312 SNP markers.

### Adjustment for the Line 1 subpopulation

The inbred population Line 1 has had a significant effect on the Hereford breed and it has been estimated that 79% of the Herefords registered from 2006 to 2008 have some proportion of Line 1 in their pedigree [33]. Due to the large number of Line 1 animals sampled in the “2,000 Bull Project” and Line 1’s general influence among the Hereford breed a second series of analyses were performed to remove the excessive population bias from the dataset. A Bayesian analysis was performed using STRUCTURE software [34] to estimate the Line 1 contribution to each of the animals in the dataset. Individuals with a posterior probability of > 0.37 in relation to Line 1 were removed from the dataset. The remaining samples (n = 246) were used to estimate pairwise coefficients of relationships, between and among the five ecoregions using existing pedigree data supplied by the American Hereford Association (AHA).

### Pedigree analysis

Pedigree records for all animals in the study were obtained from AHA. The Animal Breeder’s Tool Kit [35] was used to construct pedigree files and compute coefficients of genetic relationships. Each animal’s pedigree was traced back until an unknown ancestor, for most animals the unknown ancestor was found about 1940 when electronic files of the association stop. Animals with relationship coefficients > 0.40 and not classified in the same geographic region were removed from the dataset to further reduce genetic migration bias. Making this adjustment served to better ensure that the populations within an ecoregion were not confounded with animals outside the ecoregions.

### Ecoregional assignment

A primary goal in preparing data for the analysis was to classify cattle into their respective ecoregions. An important element was to ensure the animals analyzed had been raised in an ecoregion and their sire and dam were also from the same ecoregion. Due to the large number of Line 1 animals sampled in the “2000 Bull Project” and Line 1’s general influence among the Hereford breed a series of preliminary analyses were performed to evaluate the impact of Line 1. This included evaluating the coefficient of relationships and deleting animals with a high coefficient of relationship, and using proportional assignments made by STRUCTURE [33] to identify and delete animals that had a large genetic relationships to Line 1 (posterior probability of > 0.37). In addition to the attention paid to Line 1 animals raised outside CA we eliminated non-Line 1 animals with high coefficient genetic relationships (> 0.40) with animals from different ecoregions. After these steps had been taken there were 225 animals used in the analyses and the numbers per ecoregion were: CA = 38, CH = 21, TZ = 97, WA = 49 and WH = 20.

### Fine structure genetic analysis

ADMIXTURE 1.3 [36] was used to evaluate the fine genetic structure of Hereford animals using the 14,312 SNP markers. Ten runs were repeated for each value of *K* from 1 through 11. The estimated error observed with each K was used to determine the minimal number of clusters necessary to best explain the variation found in the analyzed samples. Graphs were plotted using the best K repetition selected by CLUMPAK software [37]. Migration rates among ecoregions were estimated considering estimated pairwise F_st_ values between the 14,312 SNP markers.

### Identification of SNPs associated to environmental and production traits

A subset of 107 SNPs previously shown to be associated with production traits in cattle (body weight, early embryonic survival, heifer conception rate, heat stress, milk yield, and productive life), and response to environmental stresses [5, 38] were used for analysis across ecoregions. Removal of duplicated SNPs resulted in a final list with 66 SNPs in the Hereford dataset and 64 SNPs in the Nelore dataset. GENALEX 6.501 [39] was used to determine the allele frequencies of SNPs in each ecoregion and Nelore dataset. Comparisons among subpopulations and breeds were performed with data in the “A/B” format, considering the “A” allele as the reference in calculations, comparing relative frequencies among populations for each specific locus.

TreeSelect [40] a program developed to differentiate selected loci within closely related populations, as used in this study was used on the 66 and 64 SNP panels. A primary feature of the software is determining if an allele frequency in a population is greater or less than the central node by forces other than genetic drift (i.e., natural selection). TreeSelect can only evaluate three populations at a time, therefore, we performed four analyses combining the ecoregions: WA-WH-TZ, CA-CH-TZ, CA-WA-TZ, and CH-WH-TZ. These combinations compare warm vs cool and wet vs dry ecoregions with the TZ being constant across the four analyses.

## Results

The approach used to estimate average monthly THI indicated that animals raised in CA and CH are generally not exposed to conditions classified as “dangerous”. However, animals raised in TZ and WA and WH are exposed to conditions in the “danger” classification for approximately 60 and 120 days per year, respectively (Fig 2).

### Population assessment

**Fig 3:**
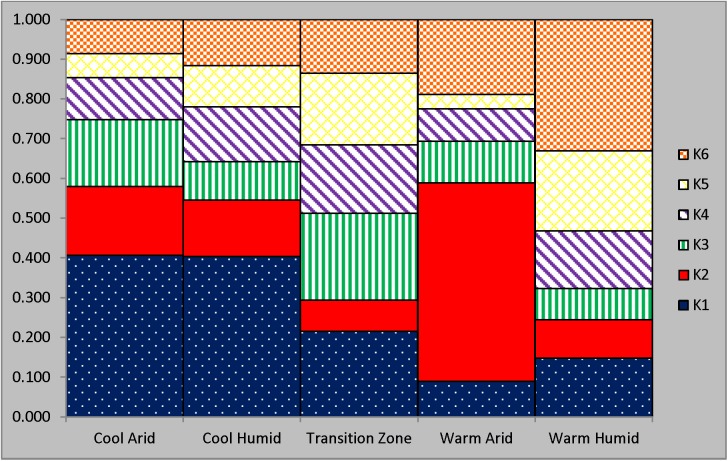
ADMIXTURE assignment of six clusters among the five ecoregions using 14,312 SNP markers.

Analysis using 14,312 SNPs revealed by the minimum estimated error generated by multiple ADMIXTURE runs was observed with K = 6 (Fig 3). In general, results reflected an association between clusters and studied ecoregions. Cluster 1 had large proportional assignments in CA and CH ecoregions (~40%). Cluster 2 assignments were more prevalent in the arid zones with a 50% assignment in WA and 17% in CA. All ecoregions were represented in clusters 3, 4 and 5. The highest and lowest proportional assignments of clusters 3, 4, and 5 were observed in TZ and WA, respectively. Cluster 6 had higher representation in WH and WA, but was present across all ecoregions in varying proportions.

Estimated migration rates among regions (Fig 1) indicate the highest level of gene flow occurs between CA and CH, corresponding to approximately 0.05% of annual registrations for the breed (Fig 1). Intermediate gene flows were found between TZ, WH, and CA. The smallest gene flows were all combinations with WA, WH-CH and WH-CA (approximately 0.01% of annual registrations). The low levels of migration observed suggest the methods used to assign animals to an ecoregion were successful.

Pedigree data supplied by AHA were used to compute average coefficients of relationship within and among the five ecoregions (Table 2) and thus provide additional insights into the breed’s genetic structure in relation to the assigned ecoregions. Observed coefficients of relationship between regions were low and ranged from 0.10 to 0.12, while slightly higher coefficients of relationship were observed within ecoregions. Observed and expected estimates of heterozygosity (Table 3) were similar among ecoregions and are in agreement with levels of heterozygosity previously reported for the breed [41].

**Table 2:** Coefficients of genetic relationship within and among ecoregions without the Line 1 subpopulation.

	Ecoregion				
Ecoregion	Cool Arid	Cool Humid	Transition Zone	Warm Arid	Warm Humid
Cool Arid	0.11				
Cool Humid	0.11	0.13			
Transition Zone	0.11	0.12	0.14		
Warm Arid	0.12	0.12	0.13	0.12	
Warm Humid	0.10	0.11	0.12	0.12	0.13

**Table 3:** Observed and expected heterozygosity estimates for Hereford cattle in five ecoregion using 14,312 SNP markers.

		Heterozygosity	
Ecoregion	Animal (n)	Observed	Expected
Cool Arid	38	0.36	0.35
Cool Humid	21	0.34	0.34
Transition Zone	97	0.34	0.35
Warm Arid	49	0.35	0.34
Warm Humid	20	0.34	0.34

### Small selected SNP panel

Establishment of a genetic substructure within a breed using the large SNP panel was followed by an evaluation of 66 selected SNPs previously reported to be associated with production traits (e.g., milk yield, body size) or ability to respond to environmental stressors (e.g., heat shock proteins).

**Fig 4:**
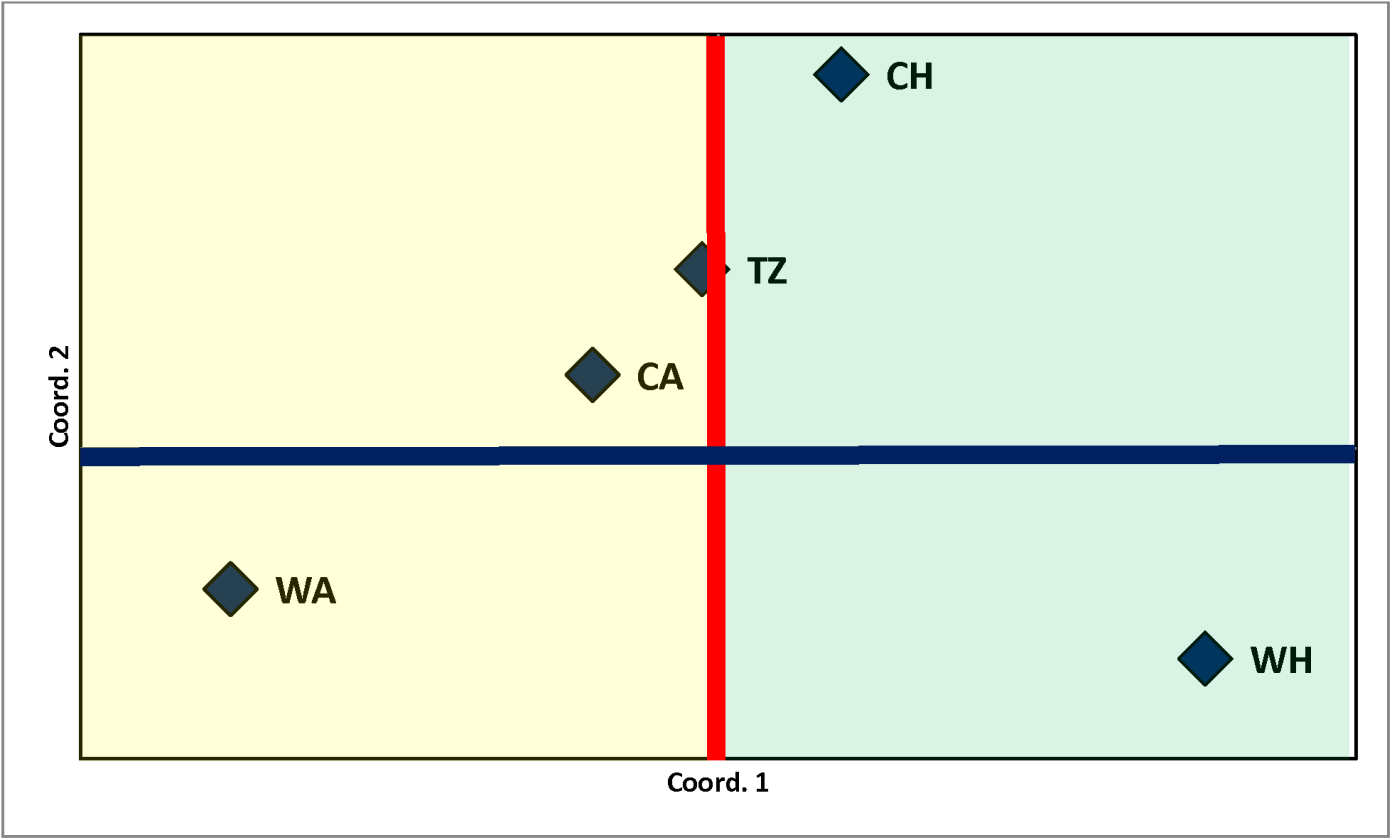
Principal coordinate analysis showing the relative positioning of cool arid (CA), cool humid (CH), transition zone (TZ), warm arid (WA) and warm humid (WH) ecoregions using 66 SNP markers.

Principal coordinate (PC) analysis with the 66 SNPs showed that the first (66.1%) and second (18.7%) PC accounted for 84.8% of the variation. Fig 4 illustrates the partitioning of ecoregions by the first and second PC along their respective midlines, and suggests that PC-1 separated the subpopulations by their presence in either arid or humid conditions. Interestingly, TZ was intermediate between the two climatic conditions. Principal component 2 partitioned subpopulations by warm vs cool temperatures.

**Fig 5:**
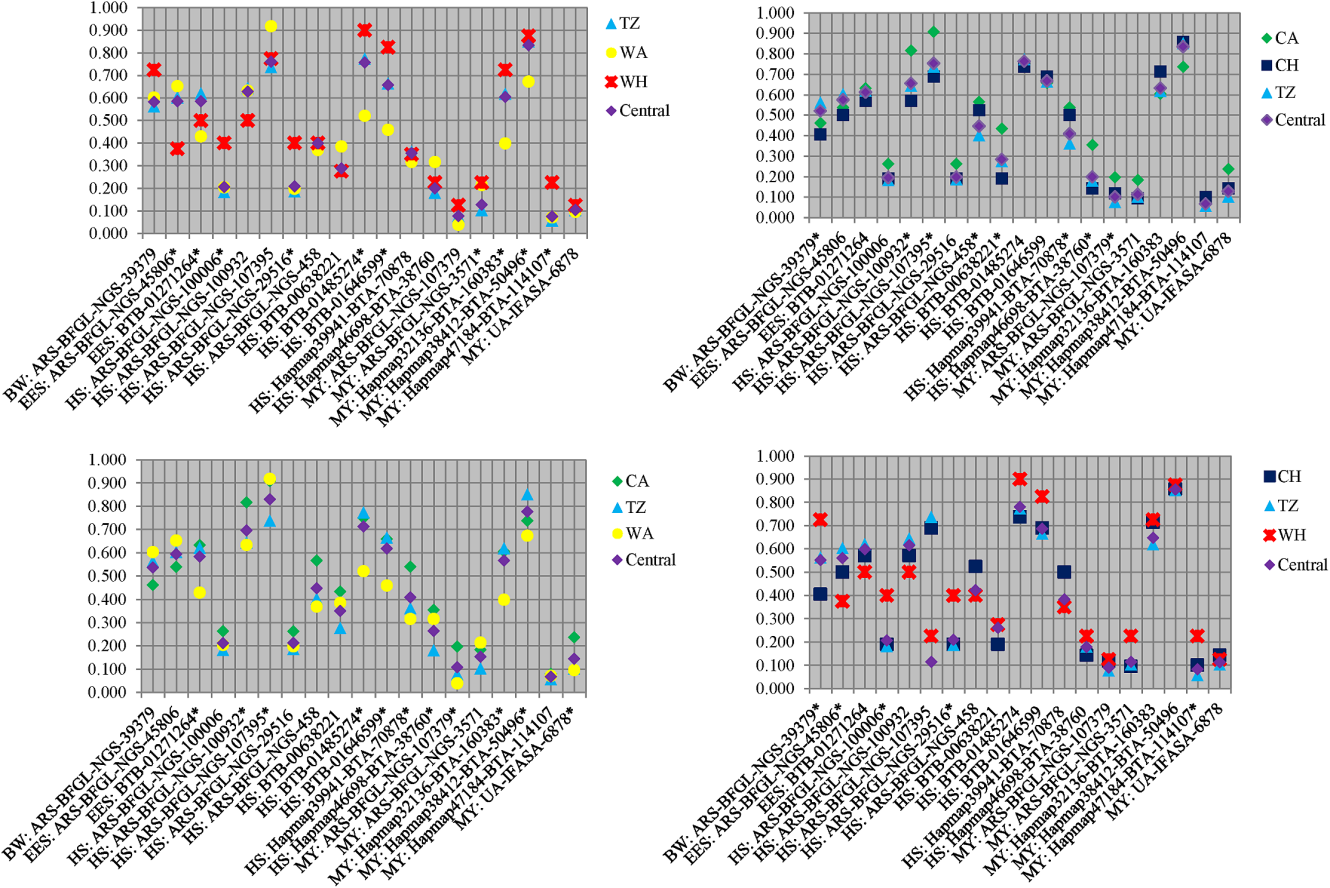
TreeSelect adjusted allele frequencies for 19 of the 66 SNPs that deviated significantly (P < 0.05) from the central allele frequency for one or more runs. Asterisk denotes significance.

TreeSelect results (Fig 5) among the four separate analysis identified 19 of the 66 SNPs as having one or more populations per run as deviating significantly (P < 0.05) from the central allele frequency. However, the 19 SNPs were not consistently significant among the four TreeSelect runs. The TZ frequency across the four analyses tended to be similar in magnitude to the computed central allele frequency, while WH, WA, CA and CH were differentiated from the central allele frequency for various SNPs among runs. The WA-WH-TZ run indicated that both WH and WA were significantly different from central allele frequency and were at opposite extremes for various SNPs associated with adaptation to environmental stressors (ARS-BFGL-NGS-45806, BTB-01271264, BTB-01485274, BTB-01646599). Differences among arid and humid populations were also observed in the CA-CH-TZ runs (ARS-BFGL-NGS-100932, ARS-BFGL-NGS-107395, BTB-00638221). For the CH-WH-TZ run a similar pattern was noted for ARS-BFGL-NGS-39379 which is associated with body weight.

**Fig 6:**
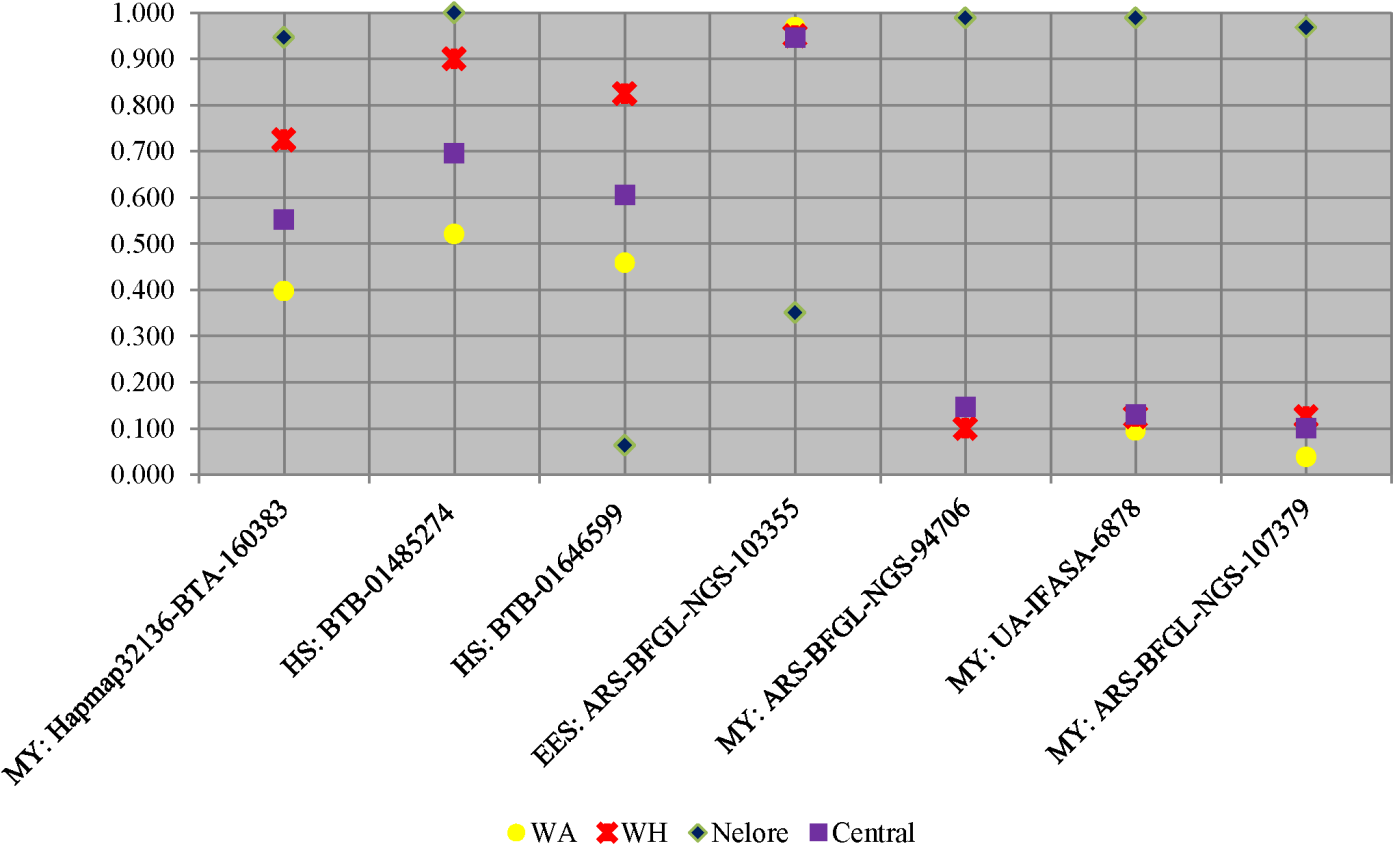
TreeSelect adjusted allele frequencies for SNPs associated with production or adaptation to environmental stress for warm arid (WA), warm humid (WH) ecoregions, and Nelore. The prefix to SNP name describes each SNP’s reported function: early embryonic survival (ESS), heifer conception rate (HCR), heat stress (HS), and milk yield (MY).

For additional perspective WA and WH subpopulations were compared to Nelore cattle with TreeSelect (Fig 6). Of the 66 SNPs only seven exhibited a population significantly different from the central allele frequency. Both Nelore and WH differed significantly from the central allele frequency for the SNPs Hapmap32136-BTA-160383, BTB-01485274, and BTB-01646599. In the remaining SNPs Nelore was the only population significantly differentiated from the central allele frequency. Comparisons of Nelore, WH and WA Hereford populations indicated the Hereford populations changed ranking in their closeness to Nelore for BTB-01485274, and BTB-01646599 SNPs associated with environmental stressors (Fig 6).

## Discussion

Climate change brings new challenges for maintaining and increasing livestock productivity. Suggestions that CC will result in changes of species and/or breeds used by livestock producers have raised awareness [7, 8]. Herefords provide an interesting model to explore the role of genetic variation and CC as the breed is found in more than 50 developed and developing countries where contrasting climates are observed [18]. Within the US the breed is widely distributed across varying climatic zones. Although comparable levels of observed and expected heterozygosity have been reported for Hereford [41] and other *Bos taurus* breeds [42], high average genetic relationships have been observed in Herefords, suggesting genetic diversity maybe more limiting than in other breeds [29, 33].

Within breed population substructures have been previously reported for cattle breeds [43], and usually result from breeding strategies intended for the development of specific genetic lines. Our results using distinct approaches and different sets of SNP markers (Bayesian and Maximum Likeihood analysis using 14K randomly distributed SNPs vs principal component analysis and TreeSelect with 66 SNPs previously shown to be associated with production traits and responses to environmental stresses) suggest Herefords could be partitioned into five subpopulations distributed across distinct ecoregions. Ecological drivers of dry vs wet conditions, as indicated by principal component analysis, or hot vs cool temperatures indicated by the Maximum Likeihood analysis may have played a significant role in changing the genetic composition of Hereford cattle evaluated in this study. Results reported here indicate a substructure that may have developed due to environmental forces due to, *inter alia*, temperature and humidity found across CA-CH, WA, WH and TZ ecoregions. Therefore, obtained results based upon molecular marker data support previous findings based on quantitative analysis of production traits which identified genetic by environmental interactions [27] and adaptation to new environments over time [5, 28] in the Hereford breed.

Estimated averages of monthly THI (Fig 2) indicate the number of days per year that Hereford subpopulations are exposed to heat stress. WA and WH have similar THI values but the allelic frequencies are different. We hypothesize this difference to be explained by additional environmental factors placing either direct or indirect selection pressure on these two sub-populations. For example, different amounts and patterns of precipitation that influence forage production; greater diurnal variation in temperature (which in the WA ecoregion may enable animals to shed heat loads more quickly [44]) and variability in the challenge of internal parasites all may play a role in the observed differences.

Analysis of pedigree data and geographic distribution of breeders across the US show that the potential for significant gene flow across ecoregions frequently is high for Herefords. Data preparation procedures were performed to minimize such confounding effects. For example, cattle with a large genetic relationship to the Line 1 and outside CA (where Line 1 was developed) were removed from the analysis, as well as, animals with large genetic relationships with animals located in different ecoregions.

Allele frequency differences observed between Nelore vs WH and WA were relatively large suggesting that while WH and WA allele frequencies approached frequencies observed in Nelore, they were not at parity (Fig 6). Despite these differences, if the evaluated SNPs are indeed indicative, obtained results suggest WH may have better capacity to adjust to hotter more humid environments [27] among the Hereford population. Differences in allele frequencies observed between WA Herefords and Nelore may suggest a different set of physiological responses are necessary when cattle are raised in hot and dry environments. This principal is corroborated by reports that heat loss and sweating rates were more affected by humidity levels in *Bos taurus* than Brahman cattle [45].

The TZ is in a central location between temperature and humidity gradients, in addition to other correlated environmental factors (Fig 1). Herefords raised in TZ have been exposed to high levels of temperature and humidity (Table 1), and have adapted to forage in pastures composed of C3 and C4 grasses. ADMIXTURE and TreeSelect analysis (Figs 3 and 5) showed that the genetic composition of cattle raised in TZ are also intermediate in comparison to the other considered ecoregions. These factors suggest that Herefords raised in TZ may be versatile in coping and adapting to various environmental conditions [46]. Herefords from TZ may have higher performance levels when compared to WA or WH cattle and therefore they may be a more desirable option for use in adapting cattle to CC conditions in some ecoregions, particularly with a selection program designed to balance improvement of productivity and adaptability [13].

These results show that a breed with a relatively high average genetic relationship and generally associated with production in temperate zones have a range of genetic variability potentially useful in their adaptation to CC. Differences between Hereford and Nelore suggest a wide range of livestock breeds may have capacity to adapt to varying climatic conditions [4]. Our results suggest that within breed selection could alter allelic frequencies associated with traits that afford animals the ability to cope with environmental vagaries.

Natural selection and/or correlated responses to selection for production characteristics are likely to allow populations within a breed to become adapted to specific environments [27]. Although a much larger number of loci are likely involved in adapting cattle to CC variables than used in the study, the range of allele frequencies observed for specific loci in subpopulations in the five evaluated ecoregions may be indicative of local optimums across a fitness landscape [47] and the presence of reaction norms [48, 49].

The presence of varying allelic frequencies in different ecoregional subpopulations suggests an array of options for breeders to use as they work to adapt their herds to CC variables. Faster responses to selection for adaption to CC may be achieved using a base population composed of animals from WA or WH ecoregions, as suggested by results obtained in the comparisons with *Bos indicus* breeds. Conversely, considering that subpopulations found within WA and WH are lower ranking in certain production traits (e.g., weaning weight, feed efficiency, etc), it may be beneficial to select from a base population formed with animals from TZ, as this subpopulation may enable a more balanced selection strategy.

This study suggest that a breed dispersed across a wide range of ecoregions may have untapped ability to respond to existing environmental differences which may potentially become increasingly larger as CC becomes more prominent. The results indicate the importance of maintaining various subpopulations within a breed to serve as reservoirs of diversity, which may be crucial for facilitating such changes.

## Supporting information

S1 Table 66 SNP and descriptions included in Hereford study (n = 225). (DOCX)

S2 Table Sixty-six SNP allele frequencies in Hereford cattle distributed to 5 U.S. ecoregions. The loci were evaluated in the “A/B” format. The “A” allele was used for frequency calculations between ecoregions at each locus.
(DOCX)
