# Supplementary material for: A fine structure genetic analysis evaluating ecoregional adaptability of a Bos taurus breed (Hereford)
Source: PLoS One. 2017 May 1;12(5):e0176474. doi: 10.1371/journal.pone.0176474 (PMC5411102; doi:10.1371/journal.pone.0176474)
Supplement: S1 Table — (DOCX) [file pone.0176474.s001.docx]

| **SNP^1^ Name** | **SNP** | **Trait** | **BTA^2^** | **Location (bp)^3^** | **Source** |
| --- | --- | --- | --- | --- | --- |
| ARS-BFGL-NGS-67327 | rs110505759 | Body Weight (mature) | 3 | 72519744 | [1] |
| ARS-BFGL-NGS-41839 | rs110564947 | Body Weight (mature) | 1 | 76416854 | [1] |
| ARS-BFGL-NGS-39379 | rs110421124 | Body Weight (mature) | 5 | 106269362 | [1] |
| ARS-BFGL-NGS-18900 | rs110059753 | Body Weight (mature) | 7 | 93218452 | [1] |
| ARS-BFGL-NGS-6079 | rs110835938 | Body Weight (mature) | 7 | 21595908 | [1] |
| ARS-BFGL-NGS-97944 | rs41711496 | Daughter Preg Rate/Prod. Life | 13 | 75567844 | [2] |
| BTB-01271264 | rs42397090 | Early Embryonic Survival | 8 | 27557552 | [3] |
| ARS-BFGL-NGS-45806 | rs110721971 | Early Embryonic Survival | 12 | 37025686 | [3] |
| ARS-BFGL-NGS-103355 | rs110464321 | Early Embryonic Survival | 13 | 19590132 | [3] |
| Hapmap58887-rs29013502 | rs29013502 | Heat Stress | 24 | 28907154 | [4] |
| Hapmap47861-BTA-120563 | rs41622115 | Heat Stress | 5 | 89472174 | [4] |
| Hapmap47403-BTA-76048 | rs41567027 | Heat Stress | 6 | 45153190 | [4] |
| Hapmap46698-BTA-38760 | rs41579673 | Heat Stress | 16 | 35317388 | [4] |
| Hapmap39941-BTA-70878 | rs41573162 | Heat Stress | 4 | 64386271 | [4] |
| Hapmap30420-BTC-039335 | rs109279094 | Heat Stress | 6 | 45175137 | [4] |
| BTB-01646599 | rs42761380 | Heat Stress | 24 | 28941584 | [4] |
| BTB-01485274 | rs42609685 | Heat Stress | 24 | 28877547 | [4] |
| BTB-01267080 | rs42394542 | Heat Stress | 5 | 89512928 | [4] |
| BTB-01267042 | rs42393904 | Heat Stress | 5 | 89568937 | [4] |
| BTB-00638221 | rs41798380 | Heat Stress | 16 | 35272426 | [4] |
| BTA-27496-no-rs | rs41609304 | Heat Stress | 12 | 2500836 | [4] |
| ARS-BFGL-NGS-89847 | rs110209659 | Heat Stress | 7 | 2457750 | [4] |
| ARS-BFGL-NGS-71584 | rs42090237 | Heat Stress | 26 | 20290497 | [4] |
| ARS-BFGL-NGS-458 | rs111023020 | Heat Stress | 4 | 64351574 | [4] |
| ARS-BFGL-NGS-41140 | rs42042561 | Heat Stress | 24 | 28975828 | [4] |
| ARS-BFGL-NGS-35716 | rs110012069 | Heat Stress | 24 | 29013292 | [4] |
| ARS-BFGL-NGS-29516 | rs109002679 | Heat Stress | 23 | 14246801 | [4] |
| ARS-BFGL-NGS-23064 | rs109890402 | Heat Stress | 26 | 20365711 | [4] |
| ARS-BFGL-NGS-16848 | rs110076378 | Heat Stress | 28 | 2924302 | [4] |
| ARS-BFGL-NGS-108847 | rs43719996 | Heat Stress | 16 | 58500249 | [4] |
| ARS-BFGL-NGS-107395 | rs110333567 | Heat Stress | 29 | 47527067 | [4] |
| ARS-BFGL-NGS-106628 | rs110691682 | Heat Stress | 16 | 35172005 | [4] |
| ARS-BFGL-NGS-10307 | rs109477915 | Heat Stress | 26 | 20259486 | [4] |
| ARS-BFGL-NGS-100932 | rs41798395 | Heat Stress | 16 | 35230105 | [4] |
| ARS-BFGL-NGS-100006 | rs41568955 | Heat Stress | 23 | 14215024 | [4] |
| ARS-BFGL-NGS-34049 | rs109262355 | Heifer Conception Rate | 20 | 35249040 | [2] |
| UA-IFASA-6878 | rs41629750 | Milk Yield | 14 | 2002873 | [5] |
| UA-IFASA-6228 | rs110718625 | Milk Yield | 14 | 6859824 | [5] |
| Hapmap32236-BTC-049785 | rs42215845 | Milk Yield | 14 | 6795860 | [5] |
| Hapmap32234-BTC-048199 | rs108995214 | Milk Yield | 14 | 7314869 | [5] |
| Hapmap30646-BTC-002054 | rs110060785 | Milk Yield | 14 | 2553525 | [5] |
| Hapmap30374-BTC-002159 | rs109529219 | Milk Yield | 14 | 2468020 | [5] |
| Hapmap30086-BTC-002066 | rs110199901 | Milk Yield | 14 | 2524432 | [5] |
| Hapmap29888-BTC-003509 | rs110237430 | Milk Yield | 14 | 2803998 | [5] |
| Hapmap26598-BTC-062212 | rs41597129 | Milk Yield | 14 | 8173920 | [5] |
| Hapmap24715-BTC-001973 | rs110323635 | Milk Yield | 14 | 2239085 | [5] |
| Hapmap23454-BTC-046932 | rs108971409 | Milk Yield | 14 | 5831267 | [5] |
| BTA-35941-no-rs | rs41627764 | Milk Yield | 14 | 2276443 | [5] |
| ARS-BFGL-NGS-94706 | rs17870736 | Milk Yield | 14 | 1696470 | [5] |
| ARS-BFGL-NGS-4939 | rs109421300 | Milk Yield | 14 | 1801116 | [5] |
| ARS-BFGL-NGS-3571 | rs110351374 | Milk Yield | 14 | 5225467 | [5] |
| ARS-BFGL-NGS-34135 | rs109968515 | Milk Yield | 14 | 1675278 | [5] |
| ARS-BFGL-NGS-26520 | rs109617015 | Milk Yield | 14 | 2386688 | [5] |
| ARS-BFGL-NGS-107379 | rs109350371 | Milk Yield | 14 | 2054457 | [5] |
| ARS-BFGL-NGS-102953 | rs110856800 | Milk Yield | 14 | 7542584 | [5] |
| ARS-BFGL-NGS-100480 | rs110017379 | Milk Yield | 14 | 4364952 | [5] |
| ARS-BFGL-BAC-24804 | rs110236070 | Milk Yield | 14 | 5795431 | [5] |
| Hapmap32136-BTA-160383 | rs110220642 | Milk Yield | 4 | 92705191 | [6] |
| Hapmap48796-BTA-51083 | rs41635833 | Milk Yield | 20 | 63120443 | [7] |
| Hapmap47184-BTA-114107 | rs41613557 | Milk Yield | 5 | 33173961 | [7] |
| Hapmap33541-BTC-016426 | rs111008794 | Milk Yield | 25 | 1431881 | [7] |
| ARS-BFGL-NGS-56044 | rs110529685 | Milk Yield | 24 | 43170091 | [7] |
| ARS-BFGL-NGS-3562 | rs109557202 | Milk Yield | 25 | 1489008 | [7] |
| Hapmap38412-BTA-50496 | rs41581070 | Milk Yield | 20 | 37468100 | [8] |
| BTA-50482-no-rs | rs41581068 | Milk Yield | 20 | 36336225 | [8] |
| BTA-37177-no-rs | rs41583256 | Milk Yield | 15 | 58775396 | [8] |
| ^1^SNP: single-nucleotide polymorphism  ^2^BTA: *Bos taurus* autosome  ^3^Location (bp): location of SNP on chromosome measured as base-pairs from the UMD3.1 assembly. | | | | | |

**Literature Cited.**

1. Saatchi M, Schnabel RD, Taylor JF, Garrick DJ (2014) Large-effect pleiotropic or closely linked QTL segregate within and across ten US cattle breeds. BMC genomics, 15(1):442. doi: 10.1186/1471-2164-15-442.
2. Cochran SD, Cole JB, Null DJ, Hansen PJ (2013) Discovery of single nucleotide polymorphisms in candidate genes associated with fertility and production traits in Holstein cattle. BMC Genet.14(1):49. doi: 10.1186/1471-2156-14-49
3. Huang W, Kirkpatrick BW, Rosa GJM, Khatib H (2010) A genome‐wide association study using selective DNA pooling identifies candidate markers for fertility in Holstein cattle. Animal Genet. 41(6):570-578.
4. Dikmen S, Cole JB, Null DJ, Hansen PJ (2013) Genome-wide association mapping for identification of quantitative trait loci for rectal temperature during heat stress in Holstein cattle. PLoS One, 8(7), p.e69202. doi: 10.1371/journal/one.0069202.
5. Meredith BK, Kearney FJ, Finlay EK, Bradley DG, Fahey AG, Berry DP, Lynn DJ (2012) Genome-wide associations for milk production and somatic cell score in Holstein-Friesian cattle in Ireland. BMC Genetic. 13(1): 21. doi: 10.1186/1471-2156-13-21
6. Maxa J, Neuditschko M, Russ I, Förster M, Medugorac I (2012) Genome-wide association mapping of milk production traits in Braunvieh cattle. J Dairy Sci. 95(9): 5357-5364. [doi:10.3168/jds.2011-4673](http://dx.doi.org/10.3168/jds.2011-4673)

7. Guo J, Jorjani H, Carlborg Ö (2012) A genome-wide association study using international breeding-evaluation data identifies major loci affecting production traits and stature in the Brown Swiss cattle breed. BMC Genet. 2: 13 (1): 82. doi:10.1186/1471-2156-13-82

8. Chamberlain AJ, Hayes BJ, Savin K, Bolormaa S, McPartlan HC, Bowman PJ, Van Der Jagt C, MacEachern S, Goddard ME. (2012) Validation of single nucleotide polymorphisms associated with milk production traits in dairy cattle. J Dairy Sci. 95(2): 865-875. doi: 10.3168/jds.2010-3786
