# Supplementary material for: A fine structure genetic analysis evaluating ecoregional adaptability of a Bos taurus breed (Hereford)
Source: PLoS One. 2017 May 1;12(5):e0176474. doi: 10.1371/journal.pone.0176474 (PMC5411102; doi:10.1371/journal.pone.0176474)
Supplement: S2 Table — The loci were evaluated in the “A/B” format. The “A” allele was used for frequency calculations between ecoregions at each locus. (DOCX) [file pone.0176474.s002.docx]

| **SNP Name** | **Cool Arid** | **Cool Humid** | **Transition Zone** | **Warm Arid** | **Warm Humid** |
| --- | --- | --- | --- | --- | --- |
| MY: ARS-BFGL-BAC-24804 | 0.289 | 0.262 | 0.366 | 0.327 | 0.200 |
| HS: ARS-BFGL-NGS-100006 | 0.737 | 0.810 | 0.818 | 0.796 | 0.600 |
| MY: ARS-BFGL-NGS-100480 | 0.554 | 0.571 | 0.624 | 0.684 | 0.575 |
| HS: ARS-BFGL-NGS-100932 | 0.184 | 0.429 | 0.356 | 0.367 | 0.500 |
| MY: ARS-BFGL-NGS-102953 | 0.145 | 0.262 | 0.196 | 0.102 | 0.100 |
| HS: ARS-BFGL-NGS-10307 | 0.158 | 0.048 | 0.072 | 0.092 | 0.125 |
| EES: ARS-BFGL-NGS-103355 | 0.013 | 0.048 | 0.021 | 0.031 | 0.050 |
| HS: ARS-BFGL-NGS-106628 | 0.342 | 0.500 | 0.526 | 0.490 | 0.550 |
| MY: ARS-BFGL-NGS-107379 | 0.803 | 0.881 | 0.923 | 0.961 | 0.875 |
| HS: ARS-BFGL-NGS-107395** | 0.092 | 0.310 | 0.263 | 0.082 | 0.225 |
| HS: ARS-BFGL-NGS-108847 | 0.500 | 0.524 | 0.459 | 0.408 | 0.375 |
| HS: ARS-BFGL-NGS-16848 | 0.105 | 0.143 | 0.094 | 0.133 | 0.050 |
| BW: ARS-BFGL-NGS-18900 | 0.579 | 0.500 | 0.634 | 0.612 | 0.550 |
| HS: ARS-BFGL-NGS-23064 | 0.842 | 0.810 | 0.820 | 0.776 | 0.800 |
| MY: ARS-BFGL-NGS-26520 | 0.197 | 0.119 | 0.206 | 0.159 | 0.200 |
| HS: ARS-BFGL-NGS-29516 | 0.737 | 0.810 | 0.814 | 0.802 | 0.600 |
| HCR: ARS-BFGL-NGS-34049 | 0.026 | 0.048 | 0.026 | 0.000 | 0.125 |
| MY: ARS-BFGL-NGS-34135 | 0.987 | 1.000 | 0.995 | 1.000 | 1.000 |
| MY: ARS-BFGL-NGS-3562 | 0.013 | 0.000 | 0.000 | 0.000 | 0.000 |
| MY: ARS-BFGL-NGS-3571 | 0.816 | 0.905 | 0.897 | 0.786 | 0.775 |
| HS: ARS-BFGL-NGS-35716 | 0.197 | 0.143* | 0.170 | 0.296 | 0.100 |
| BW: ARS-BFGL-NGS-39379 | 0.539 | 0.595 | 0.438 | 0.397* | 0.275 |
| HS: ARS-BFGL-NGS-41140 | 0.184 | 0.167 | 0.201 | 0.296 | 0.100 |
| BW: ARS-BFGL-NGS-41839 | 0.816 | 0.929 | 0.845 | 0.908 | 0.950 |
| HS: ARS-BFGL-NGS-458 | 0.434 | 0.476 | 0.598 | 0.631 | 0.600 |
| EES: ARS-BFGL-NGS-45806 | 0.461 | 0.500 | 0.397 | 0.347 | 0.625 |
| MY: ARS-BFGL-NGS-4939** | 1.000 | 1.000 | 1.000 | 1.000 | 1.000 |
| MY: ARS-BFGL-NGS-56044 | 0.776 | 0.738 | 0.758 | 0.765 | 0.800 |
| BW: ARS-BFGL-NGS-6079 | 0.724 | 0.762 | 0.639 | 0.688 | 0.750 |
| BW: ARS-BFGL-NGS-67327 | 0.171 | 0.167 | 0.242 | 0.194 | 0.250 |
| HS: ARS-BFGL-NGS-71584 | 0.895 | 0.833 | 0.868 | 0.906 | 0.725 |
| HS: ARS-BFGL-NGS-89847** | 0.000 | 0.000 | 0.000 | 0.000 | 0.000 |
| MY: ARS-BFGL-NGS-94706 | 0.895 | 0.833 | 0.845 | 0.854 | 0.900 |
| DPR/PL: ARS-BFGL-NGS-97944 | 0.487 | 0.500 | 0.412 | 0.469 | 0.525 |
| HS: BTA-27496-no-rs | 0.461 | 0.381 | 0.495 | 0.520 | 0.500 |
| MY: BTA-35941-no-rs | 0.882 | 0.810 | 0.851 | 0.847 | 0.725 |
| MY: BTA-37177-no-rs | 0.987 | 0.881 | 0.943 | 0.969 | 0.975 |
| MY: BTA-50482-no-rs | 0.526 | 0.571 | 0.588 | 0.479 | 0.650 |
| HS: BTB-00638221 | 0.566 | 0.810 | 0.724 | 0.615 | 0.725 |
| HS: BTB-01267042 | 0.816 | 0.690 | 0.722 | 0.745 | 0.800 |
| HS: BTB-01267080 | 0.368 | 0.476 | 0.448 | 0.418* | 0.425 |
| EES: BTB-01271264 | 0.368 | 0.429 | 0.380 | 0.571 | 0.500 |
| HS: BTB-01485274** | 0.250 | 0.262 | 0.227 | 0.479 | 0.100 |
| HS: BTB-01646599** | 0.342 | 0.310 | 0.335 | 0.541 | 0.175 |
| MY: Hapmap23454-BTC-046932 | 0.737 | 0.738 | 0.670 | 0.592 | 0.800 |
| MY: Hapmap24715-BTC-001973 | 0.592 | 0.619 | 0.567 | 0.765 | 0.600 |
| MY: Hapmap26598-BTC-062212 | 0.434 | 0.375 | 0.406 | 0.341 | 0.350 |
| MY: Hapmap29888-BTC-003509 | 0.434 | 0.619 | 0.562 | 0.442 | 0.550 |
| MY: Hapmap30086-BTC-002066 | 0.447 | 0.595 | 0.500 | 0.635 | 0.525 |
| MY: Hapmap30374-BTC-002159 | 0.421 | 0.405 | 0.371 | 0.272 | 0.475 |
| HS: Hapmap30420-BTC-039335 | 0.447 | 0.333 | 0.407* | 0.457 | 0.350 |
| MY: Hapmap30646-BTC-002054 | 0.395 | 0.452 | 0.392 | 0.542 | 0.425 |
| MY: Hapmap32136-BTA-160383** | 0.395 | 0.286 | 0.381 | 0.602 | 0.275 |
| MY: Hapmap32234-BTC-048199 | 0.500 | 0.595 | 0.541 | 0.459 | 0.625 |
| MY: Hapmap32236-BTC-049785 | 0.421 | 0.405 | 0.402* | 0.378 | 0.350 |
| MY: Hapmap33541-BTC-016426 | 0.395 | 0.425 | 0.351 | 0.438 | 0.325 |
| MY: Hapmap38412-BTA-50496 | 0.263 | 0.143 | 0.149 | 0.327 | 0.125 |
| HS: Hapmap39941-BTA-70878 | 0.461 | 0.500 | 0.639 | 0.684 | 0.650 |
| HS: Hapmap46698-BTA-38760 | 0.645 | 0.857 | 0.820 | 0.684 | 0.775 |
| MY: Hapmap47184-BTA-114107 | 0.921 | 0.900 | 0.943 | 0.929 | 0.775 |
| HS: Hapmap47403-BTA-76048 | 0.579 | 0.548 | 0.567 | 0.490 | 0.400 |
| HS: Hapmap47861-BTA-120563 | 0.868 | 0.857 | 0.763 | 0.888 | 0.775 |
| MY: Hapmap48796-BTA-51083 | 0.316 | 0.262 | 0.325* | 0.309 | 0.425 |
| HS: Hapmap58887-rs29013502 | 0.842 | 0.810 | 0.799 | 0.908 | 0.800 |
| MY: UA-IFASA-6228 | 0.868 | 0.976 | 0.897 | 0.908 | 0.875 |
| MY: UA-IFASA-6878 | 0.763 | 0.857 | 0.897 | 0.904 | 0.875 |
| * Significant loci (P < 0.05) under Hardy-Weinberg analysis within an ecoregion. ** Significant loci (P < 0.05) under Detection of Loci Under Selection analysis.  MY: milk yield; HS: heat stress; DPR: daughter pregnancy rate; BW: body weight; HCR: heifer conception rate; EES: early embryonic survival. | | | | | |
